# Pre‐Gestational intake of *Lactobacillus helveticus* NS8 has anxiolytic effects in adolescent Sprague Dawley offspring

**DOI:** 10.1002/brb3.1714

**Published:** 2020-07-17

**Authors:** Yunxia Niu, Shan Liang, Tao Wang, Xu Hu, Wei Li, Xiaoli Wu, Feng Jin

**Affiliations:** ^1^ School of Vocational Education Tianjin University of Technology and Education Tianjin China; ^2^ CAS Key Laboratory of Mental Health Institute of Psychology Beijing China; ^3^ Department of Psychology University of Chinese Academy of Sciences Beijing China; ^4^ CAS Key Laboratory of Microbial Physiological and Metabolic Engineering Institute of Microbiology Chinese Academy of Sciences Beijing China

**Keywords:** antianxiety, elevated plus maze test, gestation, *Lactobacillus helveticus*, open field test

## Abstract

**Introduction:**

Adolescence is a period of heightened susceptibility to anxiety disorders. Probiotic supplementation had a positive impact on reducing anxiety. The maternal microbiome plays an important role in child health outcomes and in the establishment of the offspring microbiome. Few studies have investigated the impact of gestational probiotic supplementation on the offspring's anxiety.

**Methods:**

The present study examined the impact of prenatal *Lactobacillus helveticus* NS8 supplementation (LAC) on Sprague Dawley rat offspring's anxiety‐like behavior. The behaviors tested in the present study include the elevated plus maze (EPM), the open field test (OFT), and prepulse inhibition (PPI). Analyses of variance were utilized.

**Results:**

(a) The performance of LAC adolescent rats in the EPM was similar to that in the OFT, both of which reflect that LAC caused an antianxiety effect in adolescent offspring rats and the antianxiety effect without sex differences; (b) LAC did not change performance in PPI and did not change the sex and age differences in PPI; and c. LAC decreased the body mass of rat offspring.

**Conclusion:**

*Lactobacillus helveticus* NS8 supplementation during gestation might have a moderate antianxiety effect in both males and females (especially adolescents) and be helpful for avoiding excessive body mass.

## Graphical abstract

Pregnancy *Lactobacillus helveticus* NS8 supplementation might have a moderate antianxiety effect on the offspring rats (especially adolescents) and might be helpful to avoid the excessive body mass of the offspring rats

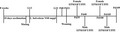

## 1 INTRODUCTION

The adolescent brain exhibits remarkable development, and adolescence can be a time of significant psychological and physiological vulnerability (Andersen, 2003; Casey, Jones, & Hare, 2008; Dahl, 2004; Kessler et al., 2007; Merikangas, 2009; Schneider, 2013; Spear, 2000). For some individuals, adolescence is a period of heightened susceptibility to anxiety disorders (Crick & Zahn‐Waxler, 2003; Giedd, Keshavan, & Paus, 2008; Petersen, 2005). Moreover, retrospective studies of children and adults suggested that childhood anxiety disorders might continue to exist for many years and could lead to other psychiatric disorders (Bland, Newman, & Orn, 1988; Burke, Burke, Regier, & Rae, 1990; Flament et al., 1988; Keller et al., 1992; Schneier, Johnson, Hornig, Liebowitz, & Weissman, 1992).

During the last few decades, the gut microbiota has become a focus of medical research and has been shown to be intertwined with various central nervous system diseases (Tremlett, Bauer, Appel‐Cresswell, Finlay, & Waubant, 2017), such as autism, major depression, schizophrenia, Alzheimer's disease, and Parkinson's disease (Dinan & Cryan, 2015; Hu, Wang, & Jin, 2016; Li et al., 2017; Sampson et al., 2016; Severance, Yolken, & Eaton, 2014). Gut microbiota might be closely related to anxiety and might provide novel approaches for prevention and treatment (Foster & Mcvey Neufeld, 2013). Gut microbiota composition is influenced by several factors, including host‐dependent factors, treatment, and diet (such as prebiotics or probiotics) (Delzenne, Neyrinck, Bäckhed, & Cani, 2011). The preclinical evidence shows that probiotic administration has some anxiolytic effects or antidepressive effects (Abildgaard, Elfving, Hokland, Lund, & Wegener, 2017; Ennio et al., 2018; Reis, Ilardi, Punt, & Jane, 2018). The clinical evidence also shows that probiotic administration has some anxiolytic effects or antidepressive effects (Eskandarzadeh et al., 2019; Lew et al., 2018; Reis et al., 2018; Slykerman et al., 2017; Pirbaglou et al., 2016).

Maternal microbes play a vital role in the establishment of the offspring microbiome (Antony et al., 2014; Collado, Rautava, Aakko, Isolauri, & Salminen, 2016; Koleva, Kim, Scott, & Kozyrskyj, 2015; Satokari, Grönroos, Laitinen, Salminen, & Isolauri, 2008) and can be transferred to the fetus (Brown et al., 2013; Paul, Bomhof, Vogel, & Reimer, 2016; Walker, Clemente, Peter, & Loos, 2017). Meanwhile, the development of the microbiome occurs simultaneously with neural development and plays a key role in neurological system maturation (Borre et al., 2014). Maternal microbiome is recognized as a key determinant of a range of maternal and child health outcomes (Dunlop et al., 2015). Evidence has shown that probiotic supplementation in early life may reduce the risk of neuropsychiatric disorders later in childhood (Pärtty, Kalliomäki, Wacklin, Salminen, & Isolauri, 2015). Evidence has also shown that probiotic supplementation in gestation is safe and might benefit gestation metabolism (Gomez‐Arango et al., 2016; Koren et al., 2012; Lindsay, Walsh, Brennan, & Mcauliffe, 2013; Luoto, Laitinen, Nermes, & Isolauri, 2010). A review study suggested that prenatal probiotic exposure might be more effective than infant exposure, influencing gut microbiota, the immune system and nutrient utilization (Naaktgeboren, 2010), and another study also indicated that microbial exposure during gestation might be even more important for its preventative effects against allergic disease (Abrahamsson, Wu, & Jenmalm, 2015).

Probiotics are defined as “live microorganisms that when administered in adequate amounts confer a health benefit on the host” (FAO & WHO, 2001). Studies have reported that many probiotics can modify the composition of gut bacteria, with a reduction in negative strains and an increase in those considered protective (Principi & Esposito, 2016). Probiotics can reduce the concentration of the harmful products of certain bacteria (Wall et al., 2012). Lactic acid bacteria are a common, effective, and safe type of probiotic (Clemente, Ursell, Parfrey, & Knight, 2012; Consortium H M P, 2012; Ljungh & Wadström, 2006; Vyas & Ranganathan, 2012). And the genus Lactobacillus is widely present in the vagina (Consortium H M P, 2012; Eloefadrosh & Rasko, 2013). And there is also one study found that pregnant *Lactobacillus rhamnosus* HN001 supplement women had significantly lower depression and anxiety scores in the postpartum period (Slykerman et al., 2017). However, few studies have investigated the effect of lactic acid bacteria supplementation during gestation and the effect of prenatal probiotic exposure on anxiety‐like behavior in offspring.

The elevated plus maze (EPM) is a validated method for assessing fear/anxiety in rats that have been used for over 30 years (Pellow, Chopin, File, & Briley, 1985). The open field test (OFT) is also one of the most widely used instruments in animal psychology and is also a rodent model of normal anxiety (Prut & Belzung, 2003). In the present study, we used the EPM and the OFT to investigate rat offspring anxiety. Prepulse inhibition (PPI) is an operational measure of sensorimotor gating or inhibition associated with frontal‐executive functioning (Perry, Minassian, Lopez, Maron, & Lincoln, 2007). PPI is a robust phenomenon that is found across species and can be studied in both humans and animals; it occurs at the first exposure and, therefore, is not a form of conditioning, and it also does not show habituation or extinction (Ellenbroek & Cools, 1990; Geyer & Markou, 1995; Koch, Garner, & Denbuuse, 2003; Swerdlow & Geyer, 1998). An increase or decrease in PPI was found to be associated with some neuropsychiatric disorders, such as schizophrenia, autism, and bipolar disorder (Braff & Geyer, 1990; Gogos, Maarten, & Rossell, 2009; Madsen, Bilenberg, Cantio, & Oranje, 2014). Thus, we plan to test PPI to evaluate sensorimotor gating.

In the present study, we aimed to evaluate the beneficial or harmful behavioral effects of lactic acid bacteria supplementation during gestation in rat offspring. *Lactobacillus helveticus* strain NS8 (*L. helveticus* strain NS8) has been shown to improve cognition in hyperammonemia rats (Luo et al., 2014) and to improve chronic restraint stress‐induced behavioral (anxiety and depression) and cognitive dysfunction in rats (Liang et al., 2015). *L. helveticus* strain NS8 was administered during the last week of gestation, which approximately coincides with the second trimester of gestation in humans, and the second trimester of gestation in humans is considered critical for vulnerability to psychiatric disorders (Bayer, Altman, Russo, & Zhang, 1993). We hypothesized that *L. helveticus* strain NS8 supplementation during gestation in rats could improve the anxiety level in the rat offspring, especially adolescent rats, without changing prepulse inhibition.

## 2 MATERIALS AND METHODS

### 2.1 Animals

Nine‐week‐old specific‐pathogen‐free (SPF) Sprague Dawley (*SD*) rats were purchased from Vital River Laboratories, including 12 female and 3 male rats. The animals were individually housed in plastic cages on a 12/12‐hr light/dark cycle (lights on at 07:00 a.m.), at a temperature ranging from 22 to 24°C and at 40%–60% humidity, with a diet of standard rodent chow and water given ad libitum at the Animal Behavior Analysis Platform of the Institute of Psychology in specific‐pathogen‐free conditions. Following 10 days of acclimation, rats were mated by a female: male ratio of 4:1. Four females were put into a male's box at 19:00 p.m. and were then placed back in their own box at 8:00 a.m. the next day. The vaginal smear method was used to identify pregnant females, and the day on which sperm in the vagina was observed was gestation 1 (G1). The female rats that mated with the same male rat were divided into different groups: control group (CON, *n* = 5) and gestational *Lactobacillus helveticus* supplement group (LAC, *n* = 4, administration *Lactobacillus helveticus* NS8 from G13 to G22). The litter size (defined as the total number of pups born per litter) ranged from 13 to 17 pups. All dams vaginally delivered their pups. Following delivery, the dam and her pups were left undisturbed in their cages until weaning on postnatal day 21 (Pd21).

According to sex and prenatal treatment, male offspring from each litter were group‐housed with three to four pups per cage, and females were placed with three or four pups per cage. According to sex, age of behavior test, and prenatal treatment, rats in the same litter were relatively uniform distributed to different sub‐groups (2–4 rats per litter). The rats were exposed to normal animal room procedures from that point forward until experimental use.

**FIGURE 1:**
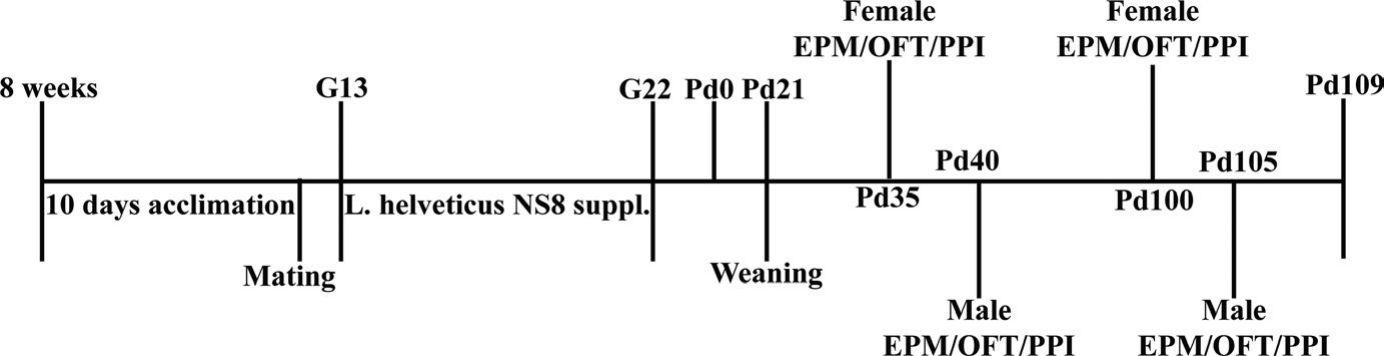
Timeline showing a summary of the experimental design. G, gestational days; L. helveticus NS8 suppl., Lactobacillus helveticus NS8 supplement; Pd, postnatal days; EPM, elevated plus maze; OFT, open field test; PPI, prepulse inhibition

Based on Schneider's review (Schneider, 2013), postnatal days 30–40 (Pd30‐40) could be considered adolescence in female rats, and postnatal days 40–50 (Pd40‐50) could be considered adolescence in male rats. Postnatal days after 60 (Pd60) could be considered adulthood in female rats and after 70 (Pd70) could be considered adulthood in male rats. Thus, here we tested the behaviors at puberty on Pd35 in the female rats (LAC, *n* = 11; CON, *n* = 11) and on Pd40 (LAC, *n* = 12; CON, *n* = 14) in the male rats; during adulthood, we tested the behaviors on Pd100 in the female rats (LAC, *n* = 14; CON, *n* = 11) and on Pd105 in the male rats (LAC, *n* = 12; CON, *n* = 12) (see Figure 1).

Body mass was measured in a pseudorandom order (the females separately on Pd28, Pd51, Pd66, Pd76, Pd86 (LAC, *n* = 14; CON, *n* = 12); the males separately on Pd28, Pd51, Pd66, Pd76, Pd86) (LAC, *n* = 12; CON, *n* = 12). An outline of the experimental design schedule is shown in Figure 1. The entire experimental protocol was approved by the Institutional Animal Care and Use Committee of the Institute of Psychology of the Chinese Academy of Sciences.

### 2.2 *Lactobacillus helveticus* NS8 strain administration

The *Lactobacillus helveticus* NS8 strain (laboratory designated numbers) was isolated from naturally fermented dairy from the Mongolia grasslands in Inner Mongolia, China P.R. The NS8 strain was inoculated into MRS media and incubated three times at 37°C for 18 hr each cycle. Then, the strain was extracted by centrifugation at 1500×*g* for 5 min and washed twice with PBS buffer. Then, the strain was resuspended in drinking water at a concentration of 10^8^ CFU/ml. The water was provided to the rats and was changed each day.

### 2.3 Behavioral testing

All behavioral testing occurred in a dedicated behavior room at the Animal Behavior Analysis Platform of the Institute of Psychology in specific‐pathogen‐free conditions. Considering the short duration of puberty and the workload factor, the present study chose to administer all behavioral tests on the same day. Behavioral assays (elevated plus maze, EPM; open field test, OFT; and prepulse inhibition, PPI) were conducted in a consistent order—1st EPM, 2nd OFT, and 3rd PPI (see Figure 1). To prevent an interaction between the tests, the time interval between tests was approximately 2 hr, and individual animals were tested in a pseudorandom order. The EPM was performed between 08:10 and 11:10, the OFT was performed between 11:10 and 13:10, and PPI was tested between 16:10 and 18:10.

#### 2.3.1 Elevated plus maze

Anxiety‐related behavior was assessed using the elevated plus maze (EPM), separately on Pd35/Pd100 in the female rats and Pd40/Pd105 in the male rats. Animals were allowed 10 min to acclimate to the testing room. The plus maze consists of two opposite open arms (50 cm × 10 cm) and two opposite closed arms (50 cm × 10 cm × 40 cm) connected by a 10 cm square center, elevated 70 cm above the floor and located in an observation room. The device is made of opaque black polypropylene. The light intensity at the end of the open arm is 10 lux, the light intensity in the double arm cross center area is 5 lux, the light intensity at the intersection of the center and the closed arm is 1–2 lux, and the intensity of the light in the closed arm is 0 lux. Rats were placed in the central area with their heads toward one corner so that they had an equal chance of going to either open arms or closed arms. An overhead camera and computer‐assisted tracking system (X‐eye Aba3.2, Beijing Macro Ambition S&T development Co. Ltd.) was used to record entries into different areas and the time spent in different areas. Following a 5 min period of maze exploration, the animal was returned to its home cage, and the maze was cleaned with 30% ethanol and dried prior to the next testing session to eliminate possible bias due to odors left by the previous rat.

A detailed factorial analysis of behavior in the plus maze revealed that the time spent in the open arms load most heavily with variables associated with anxiety‐like behavior (Cruz, Frei, & Graeff, 1994). The percentage of time spent in the open arms has usually been used as an index of fear or anxiety in many studies (Carobrez & Bertoglio, 2005). The shorter the time spent in the open arms, the higher the anxiety level is and vice versa (Ho, Eichendorff, & Schwarting, 2002; Houslay & Kolch, 2000). Thus, we analyzed the ratio of time spent in the open arms to the total time spent in all four arms as an index of anxiety‐like behavior.

#### 2.3.2 Open field test

After the EPM was completed, two hours later, the open field test (OFT) was performed. The apparatus was a circular arena, 150 cm in diameter with a 50‐cm wall, and the light intensity was 0 lux. And the test room had a dim illumination in order to decrease the averseness of the test (Sarkisova & Kulikov, 2006). The ratio of the diameter of the central area to the diameter of the circular arena is 5:13. An overhead camera and computer‐assisted tracking system (X‐eye Aba3.2, Beijing Macroambition S&T development Co. Ltd.) was used to record time and distance travelled in different areas, entries into different areas, and latency to reach the center from the peripheral part of the maze. After each animal completed the task, the apparatus was cleaned with 30% alcohol solution and was dried prior to additional testing to eliminate possible bias due to odors left by the previous rat. During each exposure, the rat was placed in the same corner, and the behavior was observed and timed for 5 min.

It has been reported that the more time that is spent in the central part and the longer the latency to central entries is, the lower the anxiety is and vice versa; additionally, the shorter the latency to reach the center from the peripheral part is, the lower the level of anxiety and vice versa (Prut & Belzung, 2003; Walsh & Cummins, 1976). In the present study, we analyzed the ratio of the time spent in the center to the total time spent in the OFT, the ratio of central part entries to total entries to the central and peripheral parts, the ratio of distance travelled in the center to the total distance travelled in the OFT, and the ratio of the latency to reach the center to the total time spent in the OFT as indices of anxiolytics.

#### 2.3.3 Prepulse inhibition of the acoustic startle reflex

Prepulse inhibition was measured with an automated startle reflex system (SR‐Lab, San Diego Instruments). The system consists of a startle chamber housed in a sound‐attenuated isolation cabinet (28.8 cm wide × 30.7 cm long × 28.5 cm high). A cylindrical holding apparatus made from transparent polypropylene (length adjusted for each rat) resting on a four‐pegged platform within the isolation chamber was used to hold each rat throughout the testing session. Below each platform, there is a piezoelectric accelerometer to detect motion. Background noise and acoustic stimuli (controlled via the SR‐Lab microcomputer and interface assembly) were delivered through a speaker mounted within the isolation chamber. All test chambers were placed in a sound‐attenuated experiment room that served to minimize external noise.

The rats were brought into the experiment room approximately a half‐hour before testing. Background noise (at 65 dB) was present throughout the testing session. After a 5‐min acclimation period to the background noise, each rat was presented with a series of 67 acoustic stimuli trials. Two types of trials were presented in a pseudorandom order. One of the types is an individual startle trial (single acoustic stimulus delivered at 115 dB for 40 ms), and the other is a prepulse stimulus trial (a single prepulse stimulus presented at 66, 68, 70, 74, 77, or 81 dB for 20 ms followed 100 ms later by a startle stimulus presented at 115 dB for 40 ms). In total, 17 individual startle trials, 7 each of the 66 and 81 dB prepulse inhibition trials, and 9 each of the 68, 70, 74, and 77 dB prepulse inhibition trials were presented. Individual startle trials were presented consecutively in groups of four at the start and end of each testing session. The average intertrial interval was 15 s. Each testing session lasted approximately 22 min. The cylindrical holding apparatus was cleaned with 30% ethanol solution between each test session. The data used for analysis are the pressures recorded by the platform floor each time the rat startles, expressed as a Vmax. The percentage of the inhibition of the startle response compared to the mean startle reflex was calculated as %PPI = 100 × (1 − mean Vmax 115 dB pulse preceded by prepulse/mean Vmax 115 dB only). Startle responses on the first and last four trials were not included in the calculation.

### 2.4 Statistical analyses

Three‐way (prenatal treatment × age ×sex) analysis of variance (ANOVA) was run for the EPM and OFT data using SPSS statistical software for Mac OSX (version 23.0), and litter size was used as a covariate.

Four‐way repeated measures analysis of variance (ANOVA) was run for prepulse inhibition data (prepulse stimulus intensity × prenatal treatment × age × sex) using SPSS statistical software for Mac OSX (version 23.0); prepulse stimulus intensity is a within‐subjects’ variable and the others are between‐subjects’ variables. Litter size was used as a covariate. BONFERRONI’s multiple comparisons correction was used as a post hoc test. Mauchly's sphericity test was significant (*p* < .01) for prepulse inhibition data. This test shows that the data do not obey the spherical hypothesis and Huynh–Feldt condition; it also shows that there are correlations between repeated measurements. It cannot be processed with one‐way ANOVA. Multivariate ANOVA is needed, and the results of the multivariate test are taken as the criteria.

Three‐way repeated measures analysis of variance (ANOVA) was run for body mass (postnatal day × prenatal treatment × sex) using SPSS statistical software for Mac OSX (version 23.0); postnatal day is a within‐subjects’ variable, and the others are between‐subjects’ variables. Litter size was used as a covariate. BONFERRONI’s multiple comparisons correction was used as a post hoc test. Mauchly's sphericity test was significant (*p* < .01) for body mass. This test shows that the data do not obey the spherical hypothesis and Huynh–Feldt condition; it also shows that there are correlations between repeated measurements. It cannot be processed with one‐way ANOVA. Multivariate ANOVA is needed, and the results of the multivariate test are taken as the criteria.

Graphics were created in GraphPad Prism6 for Mac OSX. The results are presented as averages ± standard error of the mean (*SEM*). The statistical significance level was set at *p* < .05, and a nonsignificant effect (.05 < *p* ＜ .1 was also reported in the results section.

## 3 RESULTS

### 3.1 Elevated plus maze

**FIGURE 2:**
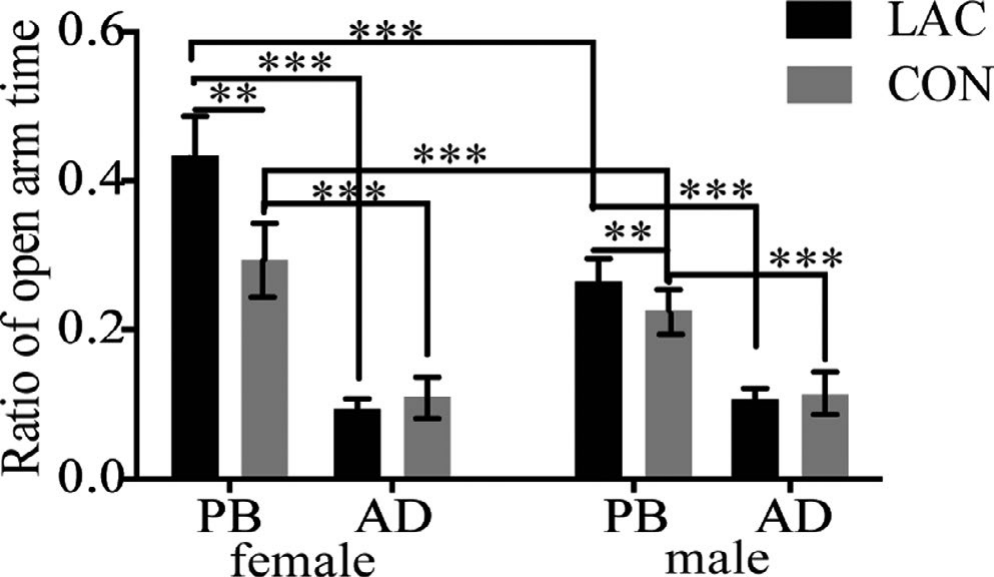
Group differences, age differences, and sex differences in the ratio of time spent in the open arms in the elevated plus maze in the rat offspring. LAC increased the ratio of open arm time in adolescent female and male rats. The ratio of open arm time in adolescent rats was significantly higher than that in adult rats. The ratio of open arm time in adolescent female rats was significantly higher than that in adolescent male rats, and there were no significant sex differences during adulthood. AD, adult; PB, puberty; CON, control group; LAC, gestational Lactobacillus helveticus NS8 supplement group; f, female; m, male. Data represent the mean ± *SEM*. Asterisks indicate significance: **p < .01, ***p < .001

For the ratio of time spent in the open arms to the total time in all four arms, a three‐way interaction between prenatal treatment, age, and sex were not observed (*F*_1,88_ = 1.49, *p* > .05, partial *η* ^2^ = 0.02), and an interaction of prenatal treatment and sex were not observed (*F*_1,88_ = 1.00, *p* > .05, partial *η* ^2^ = 0.01) (see Figure 2).

An interaction between prenatal treatment and age (*F*_1,88_ = 4.76, *p* < .05, partial *η* ^2^ = 0.05) was observed. Post hoc tests showed that the ratio of time spent in the open arms to the total time spent in all four arms in LAC adolescent rats was significantly higher than in CON adolescent rats (*p* < .01) (see Figure 2). A post hoc test also showed that the ratio of time spent in the open arms to the total time spent in all four arms in adolescent rats was significantly higher than in adult rats in both the LAC (*p* < .001) and CON (*p* < .001) groups (see Figure 2).

Meanwhile, for the ratio of time spent exploring the open arms, an interaction between sex and age (*F*_1,88_ = 7.62, *p* < .01, partial *η* ^2^ = 0.08) was observed. A post hoc test showed that the ratio of time spent in the open arms to the total time spent in all four arms in adolescent female rats was significantly higher than in adolescent male rats (*p* < .001) (see Figure 2). A post hoc test also showed that the ratio of time spent in the open arms to the total time spent in all four arms in adolescent rats was significantly higher than in adult rats in both females (*p* < .001) and males (*p* < .001) (see Figure 2).

These indicated that prenatal probiotic treatment increased the proportion of time in the open arms for both adolescent male and female rats, and the proportion of time in the open arms decreased following age, and there are sex differences in the proportion of time in the open arms for adolescent rats.

### 3.2 Open field test

**FIGURE 3:**
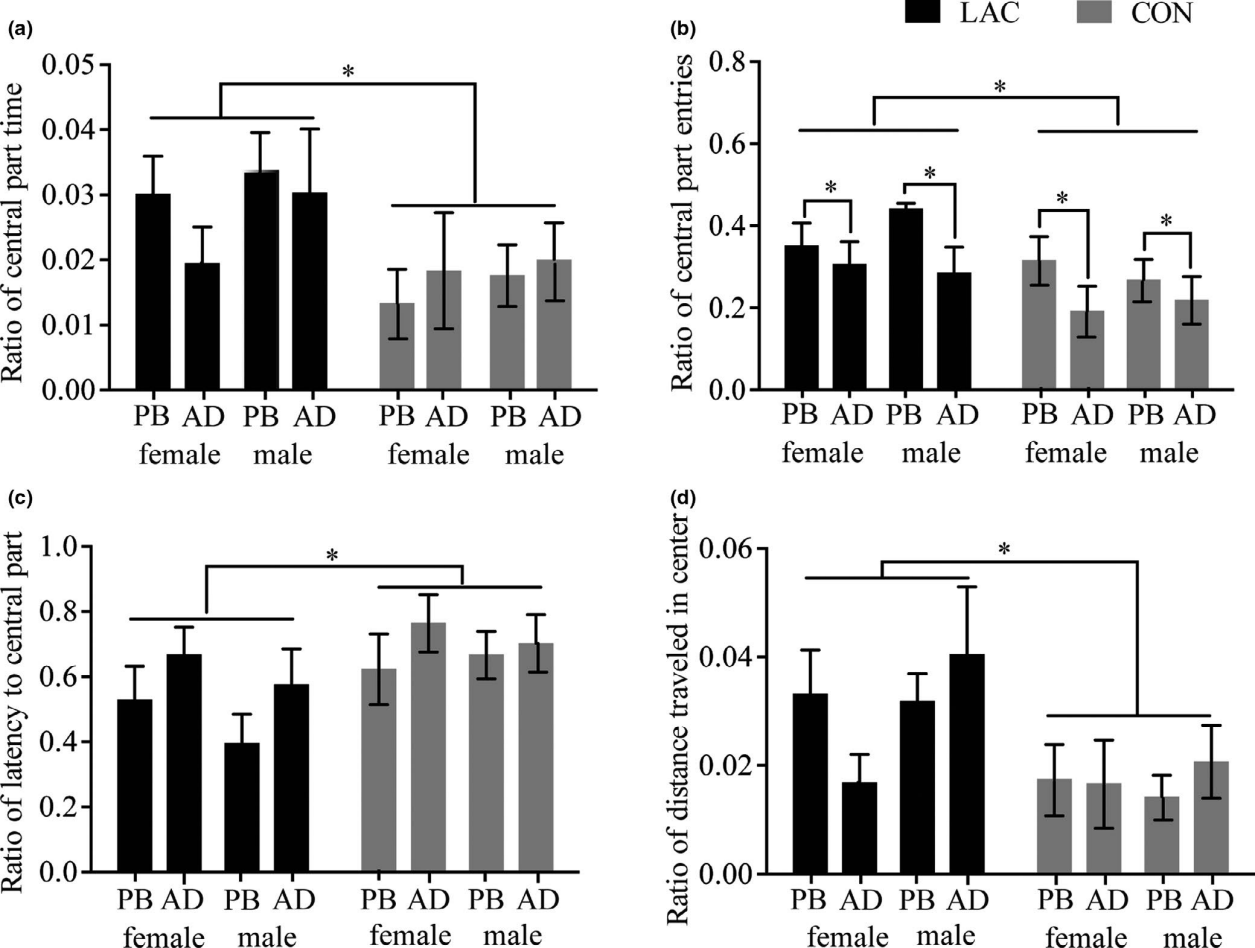
Group differences, age differences, and sex differences in the ratio of the time spent in the central part, the ratio of central part entries, the ratio of latency to enter the central part, and the ratio of distance travelled in the center in the open field test in the rat offspring. (a) and d showed that LAC increased the ratio of time spent in the central part and the ratio of distance travelled in the center in the rat offspring; sex and age did not significantly influence these indices. (b) LAC increased the ratio of central part entries; the ratio of open arm entries in adolescent female rats was significantly higher than that in adolescent male rats; and sex did not significantly influence this index. (c) LAC decreased the ratio of latency to enter central part; sex and age did not significantly influence this index. AD, adult; PB, puberty; CON, control group; LAC, gestational Lactobacillus helveticus NS8 supplement group. The data represent the mean ± *SEM*. Asterisks indicate significance: *p < .05

For the ratio of time spent in the center to the total time spent in the OFT, a main effect of prenatal treatment (*F*_1,93_ = 5.67, *p* < .05, partial *η* ^2^ = 0.06) showed that the ratio of time spent in the center to the total time spent in the OFT in the LAC group was significantly higher than that in the CON group (see Figure 3a).

For the ratio of central entries to total entries into the central and peripheral parts, main effect of prenatal treatment (*F*_1,93_ = 6.38, *p* < .05, partial *η* ^2^ = 0.06) and age (*F*_1,93_ = 6.20, *p* < .05, partial *η* ^2^ = 0.06) showed that the ratio of central entries to total entries into the central and peripheral parts in the LAC group was significantly higher than that in CON group, and the ratio of central part entries to total entries into the central and the peripheral parts in adolescent rats was significantly higher than that in adult rats (see Figure 3b).

For the ratio of the latency to reach the center from a peripheral starting position to the total time spent in the OFT, a main effect of prenatal treatment (*F*_1,93_ = 5.07, *p* < .05, partial *η* ^2^ = 0.05) showed that this ratio was significantly lower in the LAC group than in the CON group (see Figure 3c).

For the ratio of the distance travelled in the center to the total distance travelled in the OFT, a main effect of prenatal treatment (*F*_1,93_ = 6.80, *p* < .05, partial *η* ^2^ = 0.07) showed that it was significantly higher in the LAC group than in the CON group (see Figure 3d).

These indicated that prenatal probiotic treatment increased the proportion of the time/entries spent in the center and the distance travelled in the center, decreased the proportion of the latency to reach the center from the peripheral zone (in males and females during both puberty and adult stages). These also indicated that no sex‐specific differences were observed in central part entries, and the proportion of central part entries decreased with age within each sex.

### 3.3 %PPI

A four‐way interaction between prepulse intensity, prenatal treatment, age, and sex (*F*_5,86_ = 0.65, *p* = .66 > .05, partial *η* ^2^ = 0.04) was not observed. A three‐way interaction between prepulse intensity, age, and sex (*F*_5,86_ = 1.16, *p* = .33 > .05, partial *η* ^2^ = 0.06) was not observed; a three‐way interaction between prepulse intensity, prenatal treatment, and age (*F*_5,86_ = 1.42, *p* = .23 > .05, partial *η* ^2^ = 0.08) was not observed; and a three‐way interaction between prepulse intensity, prenatal treatment, and sex (*F*_5,86_ = 0.95, *p* = .45 > .05, partial *η* ^2^ = 0.05) was not observed. A three‐way interaction between prenatal treatment, age, and sex (*F*_1,90_ = 0.03, *p* = .87 > .05, partial *η* ^2^ = 0.00) was not observed.

An interaction between prepulse intensity and prenatal treatment was not observed (*F*_5,86_ = 2.04, *p* = .08 > .05, partial *η* ^2^ = 0.11); an interaction between prepulse intensity and sex was not observed (*F*_5,86_ = 1.16, *p = *.33 > .05, partial *η* ^2^ = 0.06); and an interaction between prepulse intensity and age was also not observed (*F*_5,86_ = 0.70, *p = *.62 > .05, partial *η* ^2^ = 0.04). An interaction between prenatal treatment and sex (*F*_1,90_ = 0.21, *p = *.65 > .05, partial *η* ^2^ = 0.002) was not observed. An interaction between prenatal treatment and age (*F*_1,90_ = 0.01, *p = *.94 > .05, partial *η* ^2^ = 0.00) was not observed. An interaction between sex and age (*F*_1,90_ = 0.47, *p = *.50 > .05, partial *η* ^2^ = 0.01) was not observed.

**FIGURE 4:**
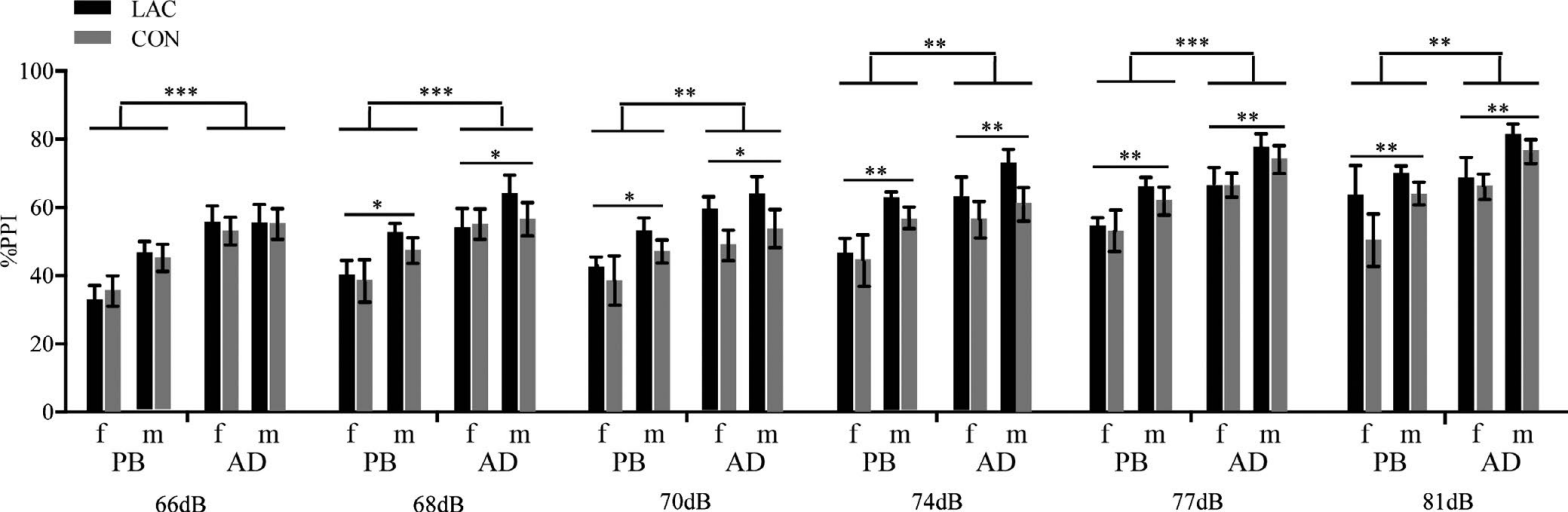
Group differences, age differences, and sex differences in %PPI in the rat offspring. There were no significant differences between CON and LAC rats in %PPI at any prepulse intensity. Adult rats exhibited higher prepulse inhibition as indicated by higher %PPI at all prepulse intensities. Male rats exhibited higher prepulse inhibition, as indicated by higher %PPI at 68, 70, 74, 77 and 81 dB prepulse intensities. %PPI, percentage of prepulse inhibition; AD, adult; PB, puberty; CON, control group; LAC, gestational Lactobacillus helveticus NS8 supplement group; dB, decibel. The data represent the mean ± *SEM*. Asterisks indicate significance: *p < .05, **p < .01, ***p < .001

A main effect of prepulse intensity (*F*_5,86_ = 1.60, *p* = .17 > .05, partial *η* ^2^ = 0.09) was not observed; a main effect of prenatal treatment (*F*_1,90_ = 1.35, *p* = .25 > .05, partial *η* ^2^ = 0.14) was not observed; a main effect of age (*F*_1,90_ = 18.21, *p* < .001, partial *η* ^2^ = 0.17) was observed; and a main effect of sex (*F*_1,90_ = 8.58, *p* < .01, partial *η* ^2^ = 0.09) was observed. BONFERRONI’s multiple comparisons correction showed that %PPI in adult rats was significantly greater than that in adolescent rats at all six prepulse intensities (66 dB, *p* < .001; 68 dB, *p* < .001; 70 dB, *p* < .01; 74 dB, *p* < .01; 77 dB, *p* < .001; 81 dB, *p* < .01) (see Figure 4). BONFERRONI’s multiple comparisons correction showed that %PPI in male rats was significantly greater than that in female rats at most prepulse intensities (68 dB, *p* < .05; 70 dB, *p* < .05; 74 dB, *p* < .01; 77 dB, *p* < .01; 81 dB, *p* < .01) (see Figure 4).

### 3.4 Body mass

A three‐way interaction of postnatal day, prenatal treatment, and sex (*F*_4,42_ = 1.40, *p* = .25 > .05, partial *η* ^2^ = 0.12) was not observed. An interaction between postnatal day and prenatal treatment was observed (*F*_4,42_ = 8.78, *p* < .001, partial *η* ^2^ = 0.46); an interaction between postnatal day and sex was also observed (*F*_4,42_ = 171.11, *p* < .001, partial *η* ^2^ = 0.94); and an interaction between prenatal treatment and sex was not significant (*F*_1,45_ = 0.13, *p* > .05, partial *η* ^2^ = 0.00). A main effect of postnatal day was observed (*F*_4,42_ = 34.46, *p* < .001, partial *η* ^2^ = 0.76), indicating that body mass increased significantly with age. A main effect of prenatal treatment was observed (*F*_1,45_ = 14.02, *p* < .01, partial *η* ^2^ = 0.24). A main effect of sex was observed (*F*_1,45_ = 371.89, *p* < .001, partial *η* ^2^ = 0.89).

**FIGURE 5:**
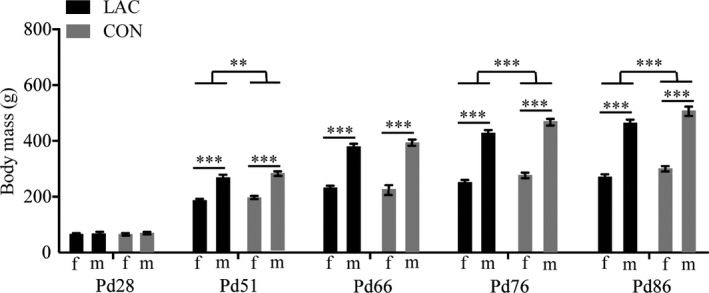
Group differences and sex differences in rat offspring body mass from Pd28 to Pd86. A. LAC rats exhibited lower body mass than CON rats on Pd51, Pd76, and Pd86. Female rats exhibited lower body mass than male rats from Pd51 to Pd86. CON, control group; LAC, gestational Lactobacillus helveticus NS8 supplement group; Pd, postnatal day. The data represent the mean ± *SEM*. Asterisks indicate significance: **p < .01, ***p < .001

BONFERRONI’s multiple comparisons correction showed that body mass in LAC rats was significantly lighter than that in CON rats on Pd51(*p* < .01), Pd76(*p* < .001), and Pd86(*p* < .001); body mass in female rats was significantly lower than that in male rats on Pd51(*p* < .001), Pd66(*p* < .001), Pd76(*p* < .001), and Pd86(*p* < .001) (see Figure 5).

## 4 DISCUSSION

### 4.1 The impact of *L. helveticus* NS8 supplementation during gestation on anxiety‐like behaviors, PPI, and body mass in rat offspring

The present study found that *L. helveticus* strain NS8 supplementation during gestation in rats could improve the anxiety level in the rat offspring (especially adolescence), without changing prepulse inhibition, which was consistent with the hypothesis. The detailed analysis is shown below.

#### 4.1.1 Anxiety‐like behaviors

The present study found that gestational *L. helveticus* NS8 supplementation decreased anxiety‐like behavior in adolescent rats in the EPM. The rats’ performance in the OFT was similar to that in the EPM, which might mean that rats’ gestational supplemented with *L. helveticus* NS8 showed significant anxiolytic effects. Studies have also found that diets containing a combination of the prebiotics polydextrose (PDX) and galactooligosaccharide (GOS) and the live bacteria *Lactobacillus rhamnosus* GG (LGG) (from weaning) attenuated the effects of early‐life maternal separation (MS) on anxiety‐like behavior (McVey Neufeld, O'Mahony, Hoban, Waworuntu, & Cryan, 2017). Notable, periconceptional antibiotic(succinylsulfathiazole) exposure has been linked with increased anxiety in offspring (Degroote, Hunting, Baccarelli, & Takser, 2016). Here, we inferred that gestational *L. helveticus* NS8 supplementation had an anxiolytic effect on the adolescent offspring rats. This result is consistent with some preclinical/ clinical evidence which shows that probiotic administration has some anxiolytic effects (Ennio et al., 2018; Eskandarzadeh et al., 2019; Lew et al., 2018; Reis et al., 2018; Slykerman et al., 2017). Future studies may consider investigating perinatal supplementation of *L. helveticus* NS8 with antibiotic treatment to attenuate postnatal anxiety symptoms in offspring during various stages of development.

The underlying mechanism is unclear. We try to make an explanation based on the existing research. It has been reported that early changes in 5‐HT homeostasis are involved in the physiopathology of psychiatric diseases, which include anxiety disorders (Ansorge, Zhou, Lira, Hen, & Gingrich, 2004; Gingrich & Hen, 2001; Gross et al., 2002; Lesch et al., 1996; Whitakerazmitia, 2001). The gastrointestinal (GI) tract contains much of the body's serotonin (5‐hydroxytryptamine, 5‐HT), and gut microbiota plays a critical role in regulating host 5‐HT levels (Yano et al., 2015). Maternal microorganisms play a vital role in the establishment of the offspring microbiome (Antony et al., 2014; Collado et al., 2016; Koleva et al., 2015; Satokari et al., 2008), and probiotic bacteria can transfer from the mother to the child (Dotterud et al., 2015). Here, *L. helveticus* NS8 strain supplementation during gestation might modify offspring gut microbiota and then improve 5‐HT homeostasis in the rat offspring, thus producing an antianxiety effect. The mechanism is worth further research.

#### 4.1.2 PPI

According to the results, we found that PPI was not influenced by gestational *L. helveticus* NS8 supplementation, and age and sex differences were also not disturbed by gestational *L. helveticus* NS8 supplementation. In addition, there was a report that periconceptional succinylsulfathiazole (a nonabsorbable antibiotic) exposure alters sensorimotor gating in rat offspring (Degroote et al., 2016). Either an increase (except the increase with age) or a decrease in PPI might reflect the aberrant sensorimotor gating that presents in some neuropsychological diseases (Braff & Geyer, 1990; Gogos et al., 2009; Hoenig, Hochrein, Quednow, Maier, & Wagner, 2005; Madsen et al., 2014; Perry et al., 2007; Swerdlow et al., 1995). Thus, we infer that either increasing or decreasing PPI is a bad thing and that gestational *L. helveticus* NS8 supplementation might not be harmful to sensorimotor gating (Perry et al., 2007). There is no research on the effect of lactic acid bacteria on PPI, so it is difficult for us to discuss this result in depth.

#### 4.1.3 Body mass

The present study found that gestational *L. helveticus* NS8 supplementation decreased the body mass of rat offspring (about from 51 days to adulthood) without influencing sex differences. Adolescence is also an especially vulnerable period for considering overweight and obesity issues in humans (Dorn & Chrousos, 1997). The present study result was also similar to a report showing that probiotic supplementation during a mother's gestation may moderate excessive weight gain in children during early childhood (Luoto et al., 2010). We inferred that *Lactobacillus helveticus* NS8 supplementation during the last week of gestation might be helpful for avoiding excessive body mass in the offspring, which supports the idea that gestational microbiota modification may offer a new direction for preventive and therapeutic applications in reducing offspring weight problems and obesity (Collado, Isolauri, Laitinen, & Salminen, 2008). On the other hand, we also infer that *Lactobacillus helveticus* NS8 supplementation disrupts the normal pattern of somatic growth. However, we did not assess feeding behavior or metabolic indices which may confound the observations.

There are also reports show body mass might be closely associated with anxiety‐like behaviors. For example, rats in the high‐fat diet showed both greater levels of anxiety‐related behavior and higher body mass (or obesity) (Noronha et al., 2016; Sivanathan, Thavartnam, Arif, Elegino, & Mcgowan, 2015). In humans, there are reports show anxiety closely associated with overweight/obesity (Namakin, Molae, Sharifzadeh, & Javadinia, 2016; Sharafi et al., 2019). And there are also reports show probiotics can modify body mass together with anxiety states in HFD (high‐fat diet)‐treated Syrian golden hamster (Ennio et al., 2018). In the present study, the lower anxiety in LAC rats might because of the lower body mass in LAC rats. Thus, gestational probiotic supplement might be a potential method to solve both the anxiety and overweight issues (especially during adolescence).

### 4.2 The differences in rat offspring anxiety‐like behaviors and PPI based on sex and age in normal environmental conditions

First, adolescent rats showed fewer anxiety‐like behaviors than adult rats in the EPM and in the OFT. This result was similar to prior studies showing that *SD* rats displayed increased anxiety‐related behavior with age (from postnatal day 74 to 346) (Ferguson & Gray, 2005) and that rats of both sexes exhibited a reduction in the time spent in the open arms of the EPM with age (between 45 and 60) (Imhof, Coelho, Schmitt, Morato, & Carobrez, 1993).

Second, adolescent female rats showed fewer anxiety‐like behaviors than adolescent male rats. This result was similar to prior studies showing that adult female *SD* rats (on postnatal day 74) stayed in the closed arms for less time than adult males (Ferguson & Gray, 2005), and adult (on postnatal 60 and 90 days) female Wistar rats and adult female Lewis rats showed fewer anxiety‐like behaviors than males (Estanislau & Morato, 2006; Imhof et al., 1993; Ramos et al., 2002). Studies showed sex differences in anxiety, both in animals and humans (Becker et al., 2005; Donner & Lowry, 2013; Johnston & File, 1991). Thus, the sex difference of anxiety‐like behavior might be cross‐variety consistency. The difference was that there were no significant sex differences between the adult rats in this study.

Third, we found that adult rats showed higher PPI than adolescent rats, and this result was similar to human studies showing that PPI demonstrated an inverted U‐shaped function with age (highest PPI at intermediate ages) (Ellwanger, Geyer, & Braff, 2003). We found that male rats showed higher PPI than females, which was in line with human studies (Blumenthal & Gescheider, 1987; Swerdlow, 1993). Then, the results can be extrapolated to humans to some extent.

There are some limitations to this study. For example, (a) we did not have a heat‐killed probiotic group that can help us determine whether live bacteria are at work or whether metabolites of bacteria are at work. (b) We also did not test the physiological indexes, like Dopamine (DA) and 5‐hydroxytryptamine (5‐HT), which are closely related to emotion; and maternal and offspring fecal sequencing, which is becoming a valuable future biomarker. (c) In experimental design, all the behavior testing was completed in one day and a consistent order, which was a potential limitation for scientific rigor. Thus, more studies are needed to support the present conclusion. There are also some interesting points that are worth studying, such as whether there are sex differences in the antianxiety effect of lactic acid bacteria, and if not, why. It would also be interesting to understand why the antianxiety effect does not continue to adulthood or how the antianxiety effect could continue to adulthood.

## 5 CONCLUSION

We found that *L. helveticus* NS8 strain supplementation during gestation has an antianxiety effect in rat offspring (especially in adolescence) and decreased the body mass of rat offspring without influencing sex differences. Meanwhile, *L. helveticus* NS8 strain supplementation during gestation did not change the sex and age differences in anxiety level, and the antianxiety effect did not increase in adulthood (and also did not weaken in adulthood); *L. helveticus* NS8 strain supplementation during gestation did not influence sex‐ and age‐dependent or independent differences in PPI. Thus, this antianxiety effect might be moderate and safe. The conclusions suggest that the maternal microbiome during gestation could be a target for modification, supporting the idea of healing the mother and healing the baby to some extent.

## CONFLICT OF INTEREST

The authors declare that they have no competing interests.

## AUTHORS’ CONTRIBUTIONS

Jin and Niu conceived and designed the study. Niu, Liang, Wang, Li, Hu, and Wu performed the experiments. Niu wrote the paper. Niu, Liang, Wang, Li, Hu, and Jin were involved in revising it critically. Jin and Niu participated in the design and coordination of the paper. All authors read and approved the manuscript and agree to be accountable for all aspects of the work.

## Data Availability

The data that support the findings of this study are available from the corresponding author upon reasonable request.

## References

[brb31714-bib-0001] Abildgaard, A. , Elfving, B. , Hokland, M. , Lund, S. , & Wegener, G. (2017). Probiotic treatment protects against the pro‐depressant‐like effect of high‐fat diet in flinders sensitive line rats. Brain, Behavior, and Immunity, 65, 33–42. 10.1016/j.bbi.2017.04.017 28450222

[brb31714-bib-0002] Abrahamsson, T. R. , Wu, R. Y. , & Jenmalm, M. C. (2015). Gut microbiota and allergy: The importance of the pregnancy period. Pediatric Research, 77(1–2), 214–219. 10.1038/pr.2014.165 25310761

[brb31714-bib-0003] Andersen, S. L. (2003). Trajectories of brain development: Point of vulnerability or window of opportunity. Neuroscience and Biobehavioral Reviews, 27(1–2), 3–18. 10.1016/S0149-7634(03)00005-8 12732219

[brb31714-bib-0004] Ansorge, M. S. , Zhou, M. , Lira, A. , Hen, R. , & Gingrich, J. A. (2004). Early‐life blockade of the 5‐HT transporter alters emotional behavior in adult mice. Science, 306(5697), 879–881. 10.1126/science.1101678 15514160

[brb31714-bib-0005] Antony, A. K. , Aagaard, K. , Ganu, R. S. , Versalovic, J. , Petrosino, J. F. , & Ma, J. (2014). The placenta harbors a unique microbiome. Science Translational Medicine, 6(237), 237ra65 10.1126/scitranslmed.3008599 PMC4929217 24848255

[brb31714-bib-0006] Avolio, E. , Fazzari, G. , Zizza, M. , De Lorenzo, A. , Di Renzo, L. , Alò, R. , … Canonaco, M. (2018). Probiotics modify body weight together with anxiety states via pro‐inflammatory factors in hfd‐treated syrian golden hamster. Behavioural Brain Research, 356, 390–399. 10.1016/j.bbr.2018.09.010 30223002

[brb31714-bib-0007] Bayer, S. A. , Altman, J. , Russo, R. J. , & Zhang, X. (1993). Timetables of neurogenesis in the human brain based on experimentally determined patterns in the rat. Neurotoxicology, 14(1), 83–144. 10.1016/0028-3908(93)90118-M 8361683

[brb31714-bib-0008] Becker, J. B. , Arnold, A. P. , Berkley, K. J. , Blaustein, J. D. , Eckel, L. A. , Hampson, E. , … Young, E. (2005). Strategies and methods for research on sex differences in brain and behavior. Endocrinology, 146(4), 1650–1673. 10.1210/en.2004-1142 15618360

[brb31714-bib-0009] Bland, R. C. , Newman, S. C. , & Orn, H. (1988). Age of onset of psychiatric disorder. Acta Psychiatrica Scandinavica Supplementum, 338(suppl), 43–49. 10.1111/j.1600-0447.1988.tb08546.x 3165594

[brb31714-bib-0010] Blumenthal, T. , & Gescheider, G. (1987). Modification of the acoustic startle reflex by a tactile prepulse: The effects of stimulus onset asynchrony and prepulse intensity. Psychophysiology, 24(3), 320–327. 10.1111/j.1469-8986.1987.tb00302.x 3602288

[brb31714-bib-0011] Borre, Y. E. , O’Keeffe, G. W. , Clarke, G. , Stanton, C. , Dinan, T. G. , & Cryan, J. F. (2014). Microbiota and neurodevelopmental windows: Implications for brain disorders. Trends in Molecular Medicine, 20(9), 509–518. 10.1016/j.molmed.2014.05.002 24956966

[brb31714-bib-0012] Braff, D. L. , & Geyer, M. A. (1990). Sensorimotor gating and schizophrenia: Human and animal model studies. Archives of General Psychiatry, 47(2), 181–188. 10.1001/archpsyc.1990.01810140081011 2405807

[brb31714-bib-0013] Brown, J. , de Vos, W. M. , DiStefano, P. S. , Doré, J. , Huttenhower, C. , Knight, R. , … Turnbaugh, P. (2013). Translating the human microbiome. Nature Biotechnology, 31(4), 304–308. 10.1038/nbt.2543 23563424

[brb31714-bib-0014] Burke, K. C. , Burke, J. D. , Regier, D. A. , & Rae, D. S. (1990). Age at onset of selected mental disorders in five community populations. Archives of General Psychiatry, 47(6), 511–518. 10.1001/archpsyc.1990.01810180011002 2350203

[brb31714-bib-0015] Carobrez, A. P. , & Bertoglio, L. J. (2005). Ethological and temporal analyses of anxiety‐like behavior: The elevated plus‐maze model 20 years on. Neuroscience & Biobehavioral Reviews, 29(8), 1193–1205. 10.1016/j.neubiorev.2005.04.017 16084592

[brb31714-bib-0016] Casey, B. J. , Jones, R. M. , & Hare, T. A. (2008). The adolescent brain. Annals of the New York Academy of Sciences, 28(1), 62–77. 10.1016/j.dr.2007.08.003 PMC2475802 18400927

[brb31714-bib-0017] Clemente, J. , Ursell, L. , Parfrey, L. , & Knight, R. (2012). The impact of the gut microbiota on human health: An integrative view. Cell, 148(6), 10.1016/j.cell.2012.01.035 PMC5050011 22424233

[brb31714-bib-0018] Collado, M. C. , Isolauri, E. , Laitinen, K. , & Salminen, S. (2008). Distinct composition of gut microbiota during pregnancy in overweight and normal‐weight women. The American Journal of Clinical Nutrition, 88(4), 894–899. 10.1093/ajcn/88.4.894 18842773

[brb31714-bib-0019] Collado, M. C. , Rautava, S. , Aakko, J. , Isolauri, E. , & Salminen, S. (2016). Human gut colonisation may be initiated in utero by distinct microbial communities in the placenta and amniotic fluid. Scientific Reports, 6, 23129 10.1038/srep23129 27001291 PMC4802384

[brb31714-bib-0020] Consortium H M P (2012). Structure, function and diversity of the healthy human microbiome. Nature, 486(7402), 207–214. 10.1038/nature11234 22699609 PMC3564958

[brb31714-bib-0021] Crick, N. R. , & Zahn‐Waxler, C. (2003). The development of psychopathology in females and males: Current progress and future challenges. Development and Psychopathology, 15(3), 719–742. 10.1017/S095457940300035X 14582938

[brb31714-bib-0022] Cruz, A. P. M. , Frei, F. , & Graeff, F. G. (1994). Ethopharmacological analysis of rat behavior on the elevated plus‐maze. Pharmacology Biochemistry & Behavior, 49(1), 171–176. 10.1007/s00441-013-1581-2 7816869

[brb31714-bib-0023] Dahl, R. E. (2004). Adolescent brain development: A period of vulnerabilities and opportunities. keynote address. Annals of the New York Academy of Sciences, 1021(1), 1–22. 10.1196/annals.1308.001 15251869

[brb31714-bib-0024] de Noronha, S. R. , Campos, G. V. , Abreu, A. R. , de Souza, A. A. , Chianca, D. A. , & de Menezes, R. C. (2016). High fat diet induced‐obesity facilitates anxiety‐like behaviors due to gabaergic impairment within the dorsomedial hypothalamus in rats. Behavioural Brain Research, 316, 38–46. 10.1016/j.bbr.2016.08.042 27566182

[brb31714-bib-0025] Degroote, S. , Hunting, D. J. , Baccarelli, A. A. , & Takser, L. (2016). Maternal gut and fetal brain connection: Increased anxiety and reduced social interactions in Wistar rat offspring following peri‐conceptional antibiotic exposure. Progress in Neuro‐Psychopharmacology and Biological Psychiatry, 71, 76–82. 10.1016/j.pnpbp.2016.06.010 27346743 PMC6584952

[brb31714-bib-0026] Delzenne, N. M. , Neyrinck, A. M. , Bäckhed, F. , & Cani, P. D. (2011). Targeting gut microbiota in obesity: Effects of prebiotics and probiotics. Nature Reviews Endocrinology, 7, 639–746. 10.1038/nrendo.2011.126 21826100

[brb31714-bib-0099] Dinan Timothy G. , Cryan John F. (2015). The impact of gut microbiota on brain and behaviour. Current Opinion in Clinical Nutrition and Metabolic Care, 18(6), 552–558. 10.1097/mco.0000000000000221 26372511

[brb31714-bib-0027] Donner, N. C. , & Lowry, C. A. (2013). Sex differences in anxiety and emotional behavior. Pflugers Archiv ‐ European Journal of Physiology, 465(5), 601–626. 10.1007/s00424-013-1271-7 23588380 PMC3805826

[brb31714-bib-0028] Dorn, L. D. , & Chrousos, G. P. (1997). The Neurobiology of stress: Understanding regulation of affect during female biological transitions. Seminars in Reproductive Medicine, 15(01), 19–36. 10.1055/s-2008-1067965 9065975

[brb31714-bib-0100] Dotterud Christian K. , Avershina Ekaterina , Sekelja Monika , Simpson Melanie R. , Rudi Knut , Storrø Ola , Johnsen Roar , Øien Torbjørn (2015). Does Maternal Perinatal Probiotic Supplementation Alter the Intestinal Microbiota of Mother and Child?. Journal of Pediatric Gastroenterology and Nutrition, 61, (2), 200–207. 10.1097/mpg.0000000000000781 25782657

[brb31714-bib-0029] Dunlop, A. L. , Mulle, J. G. , Ferranti, E. P. , Edwards, S. , Dunn, A. B. , & Corwin, E. J. (2015). Maternal microbiome and pregnancy outcomes that impact infant health: A Review. Advances in Neonatal Care, 15(6), 377 10.1097/ANC.0000000000000218 26317856 PMC4658310

[brb31714-bib-0030] Ellenbroek, B. A. , & Cools, A. R. (1990). Animal models with construct validity for schizophrenia. Behavioural Pharmacology, 1(6), 469–490. 10.1097/00008877-199000160-00001 11175433

[brb31714-bib-0031] Ellwanger, J. , Geyer, M. A. , & Braff, D. L. (2003). The relationship of age to prepulse inhibition and habituation of the acoustic startle response. Biological Psychology, 62(3), 175–195. 10.1016/S0301-0511(02)00126-6 12633977

[brb31714-bib-0032] Eloefadrosh, E. A. , & Rasko, D. A. (2013). The human microbiome: From symbiosis to pathogenesis. Annual Review of Medicine, 64(64), 145–163. 10.1146/annurev-med-010312-133513 PMC3731629 23327521

[brb31714-bib-0033] Eskandarzadeh, S. , Effatpanah, M. , Khosravi‐Darani, K. , Askari, R. , Hosseini, A. F. , Reisian, M. , & Jazayeri, S. (2019). Efficacy of a multispecies probiotic as adjunctive therapy in generalized anxiety disorder: A double blind, randomized, placebo‐controlled trial. Nutritional Neuroscience, 10.1080/1028415X.2019.1598669 31516094

[brb31714-bib-0034] Estanislau, C. , & Morato, S. (2006). Behavior ontogeny in the elevated plus‐maze: Prenatal stress effects. International Journal of Developmental Neuroscience, 24(4), 255–262. 10.1016/j.ijdevneu.2006.03.001 16698220

[brb31714-bib-0035] FAO , & WHO (2001). . Report of a joint FAO/WHO expert consultation on evaluation of health and nutritional properties of probiotics in food including powder milk with live lactic acid bacteria.. .

[brb31714-bib-0036] Ferguson, S. A. , & Gray, E. P. (2005). Aging effects on elevated plus maze behavior in spontaneously hypertensive, wistar‐kyoto and sprague‐dawley male and female rats. Physiology & Behavior, 85(5), 621–628. 10.1016/j.physbeh.2005.06.009 16043200

[brb31714-bib-0037] Flament, M. F. , Whitaker, A. , Rapoport, J. L. , Davies, M. , Berg, C. Z. , Kalikow, K. , … Shaffer, D. (1988). Obsessive compulsive disorder in adolescence: An epidemiological study. Journal of the American Academy of Child & Adolescent Psychiatry, 27(6), 764–771. 10.1097/00004583-198811000-00018 3264280

[brb31714-bib-0038] Foster, J. A. , & Mcvey Neufeld, K. A. (2013). Gut–brain axis: How the microbiome influences anxiety and depression. Trends in Neurosciences, 36(5), 305–312. 10.1016/j.tins.2013.01.005 23384445

[brb31714-bib-0039] Geyer, M. A. , & Markou, A. (1995). Animal Models of Psychiatric Disorders In Bloom F. E., & Kupfer D. J. (Eds.), Psychopharmacology: The fourth generation of progress (pp. 787–798). New York: Raven Press.

[brb31714-bib-0040] Giedd, J. N. , Keshavan, M. , & Paus, T. (2008). Why do many psychiatric disorders emerge during adolescence ? Nature Reviews Neuroscience, 9(12), 947–957. 10.1038/nrn2513 19002191 PMC2762785

[brb31714-bib-0041] Gingrich, J. A. , & Hen, R. (2001). Dissecting the role of the serotonin system in neuropsychiatric disorders using knockout mice. Psychopharmacology (Berl), 155(1), 1–10. 10.1007/s002130000573 11374326

[brb31714-bib-0042] Gogos, A. , Maarten, V. D. B. , & Rossell, S. (2009). Gender differences in prepulse inhibition (PPI) in bipolar disorder: Men have reduced PPI, women have increased PPI. The International Journal of Neuropsychopharmacology, 12(09), 1249 10.1017/S1461145709000480 19490735

[brb31714-bib-0043] Gomez‐Arango, L. F. , Barrett, H. L. , Mcintyre, H. D. , Callaway, L. K. , Morrison, M. , & Nitert, M. D. (2016). Connections between the gut microbiome and metabolic hormones in early pregnancy in overweight and obese women. Diabetes, 65(8), 2214 10.2337/db16-0278 27217482

[brb31714-bib-0044] Gross, C. , Zhuang, X. , Stark, K. , Ramboz, S. , Oosting, R. , Kirby, L. , … Hen, R. (2002). Serotonin1a receptor acts during development to establish normal anxiety‐like behaviour in the adult. Nature (London), 416(6879), 396–400. 10.1038/416396a 11919622

[brb31714-bib-0045] Ho, Y. J. , Eichendorff, J. , & Schwarting, R. K. (2002). Individual response profiles of male wistar rats in animal models for anxiety and depression. Behavioural Brain Research, 136(1), 1–12. 10.1016/S0166-4328(02)00089-X 12385785

[brb31714-bib-0046] Hoenig, K. , Hochrein, A. , Quednow, B. B. , Maier, W. , & Wagner, M. (2005). Impaired prepulse inhibition of acoustic startle in obsessive‐compulsive disorder. Biological Psychiatry, 57(10), 1153–1158. 10.1016/j.biopsych.2005.01.040 15866555

[brb31714-bib-0047] Houslay, M. D. , & Kolch, W. (2000). Cell‐type specific integration of cross‐talk between extracellular signal‐regulated kinase and camp signaling. Molecular Pharmacology, 58(4), 659–668. 10.1124/mol.58.4.659 10999934

[brb31714-bib-0048] Hu, X. , Wang, T. , & Jin, F. (2016). Alzheimer's disease and gut microbiota. Science China‐Life Sciences, 59(10), 1006–1023. 10.1007/s11427-016-5083-9 27566465

[brb31714-bib-0049] Imhof, J. T. , Coelho, Z. M. , Schmitt, M. L. , Morato, G. S. , & Carobrez, A. P. (1993). Influence of gender and age on performance of rats in the elevated plus maze apparatus. Behavioural Brain Research, 56(2), 177–180. 10.1016/0166-4328(93)90036-P 8240712

[brb31714-bib-0050] Johnston, A. L. , & File, S. E. (1991). Sex differences in animal tests of anxiety. Physiology and Behavior, 49(2), 245–250. 10.1016/0031-9384(91)90039-Q 2062894

[brb31714-bib-0051] Keller, M. B. , Lavori, P. W. , Wunder, J. , Beardslee, W. R. , Schwartz, C. E. , & Roth, J. (1992). Chronic course of anxiety disorders in children and adolescents. Journal of the American Academy of Child & Adolescent Psychiatry, 31(4), 595–599. 10.1097/00004583-199207000-00003 1644719

[brb31714-bib-0052] Kessler, R. C. , Amminger, G. P. , Aguilar‐Gaxiola, S. , Alonso, J. , Lee, S. , & Ustun, T. (2007). Age of onset of mental disorders: A review of recent literature. Current Opinion in Psychiatry, 20(4), 359–364. 10.1097/YCO.0b013e32816ebc8c 17551351 PMC1925038

[brb31714-bib-0053] Koch, M. , Garner, B. , & Denbuuse, M. V. (2003). Neurodevelopmental animal models of schizophrenia: Effects on prepulse inhibition. Current Molecular Medicine, 3(5), 459–471. 10.2174/1566524033479627 12942999

[brb31714-bib-0054] Koleva, P. T. , Kim, J. S. , Scott, J. A. , & Kozyrskyj, A. L. (2015). Microbial programming of health and disease starts during fetal life. Birth Defects Research Part C: Embryo Today: Reviews, 105(4), 265–277. 10.1002/bdrc.21117 26663884

[brb31714-bib-0055] Koren, O. , Goodrich, J. K. , Cullender, T. C. , Spor, A. , Laitinen, K. , Kling Bäckhed, H. , … Ley, R. E. (2012). Host remodeling of the gut microbiome and metabolic changes during pregnancy. Cell, 150(3), 470–480. 10.1016/j.cell.2012.07.008 22863002 PMC3505857

[brb31714-bib-0056] Lesch, K.‐P. , Bengel, D. , Heils, A. , Sabol, S. Z. , Greenberg, B. D. , Petri, S. , … Murphy, D. L. (1996). Association of anxiety‐related traits with a polymorphism in the serotonin transporter gene regulatory region. Science (Washington D C), 274(5292), 1527–1531. 10.1126/science.274.5292.1527 8929413

[brb31714-bib-0057] Lew, L.‐C. , Hor, Y.‐Y. , Yusoff, N. A. A. , Choi, S.‐B. , Yusoff, M. S. B. , Roslan, N. S. , … Liong, M.‐T. (2018). Probiotic Lactobacillus plantarum P8 alleviated stress and anxiety while enhancing memory and cognition in stressed adults: A randomised, double‐blind, placebo‐controlled study. Clinical Nutrition, 38(5), 2053–2064. 10.1016/j.clnu.2018.09.010 30266270

[brb31714-bib-0058] Li, W. , Wu, X. , Hu, X. U. , Wang, T. , Liang, S. , Duan, Y. , … Qin, B. (2017). Structural changes of gut microbiota in parkinson's disease and its correlation with clinical features. Science China‐Life Sciences, 60(11), 1223–1233. 10.1007/s11427-016-9001-4 28536926

[brb31714-bib-0059] Liang, S. , Wang, T. , Hu, X. , Luo, J. , Li, W. , Wu, X. , … Jin, F. (2015). Administration of lactobacillus helveticus ns8 improves behavioral, cognitive, and biochemical aberrations caused by chronic restraint stress. Neuroscience, 310(21), 561–577. 10.1016/j.neuroscience.2015.09.033 26408987

[brb31714-bib-0060] Lindsay, K. L. , Walsh, C. A. , Brennan, L. , & Mcauliffe, F. M. (2013). Probiotics in pregnancy and maternal outcomes: A systematic review. Journal of Maternal‐Fetal Medicine, 26(8), 772–778. 10.3109/14767058.2012.755166 23205866

[brb31714-bib-0061] Ljungh, A. , & Wadström, T. (2006). Lactic acid bacteria as probiotics. Current Issues in Intestinal Microbiology, 7(2), 73–89. 10.1111/j.1574-6968.2010.02185.x 16875422

[brb31714-bib-0062] Luo, J. , Wang, T. , Liang, S. , Hu, X. , Li, W. , & Jin, F. (2014). Ingestion oflactobacillusstrain reduces anxiety and improves cognitive function in the hyperammonemia rat. Science China Life Sciences, 57(3), 327–335. 10.1007/s11427-014-4615-4 24554471

[brb31714-bib-0063] Luoto, R. , Laitinen, K. , Nermes, M. , & Isolauri, E. (2010). Impact of maternal probiotic‐supplemented dietary counselling on pregnancy outcome and prenatal and postnatal growth: A double‐blind, placebo‐controlled study. British Journal of Nutrition, 103(12), 1792–1799. 10.1017/S0007114509993898 20128938

[brb31714-bib-0064] Madsen, G. F. , Bilenberg, N. , Cantio, C. , & Oranje, B. (2014). Increased prepulse inhibition and sensitization of the startle reflex in autistic children. Autism Research, 7(1), 94–103. 10.1002/aur.1337 24124111

[brb31714-bib-0065] McVey Neufeld, K. A. , O'Mahony, S. M. , Hoban, A. E. , Waworuntu, R. V. , & Cryan, J. F. (2017). Neurobehavioural effects of lactobacillus rhamnosus gg alone and in combination with prebiotics polydextrose and galactooligosaccharide in male rats exposed to early‐life stress. Nutritional Neuroscience, 4, 1–10. 10.1080/1028415X.2017.1397875 29173065

[brb31714-bib-0066] Merikangas, K. (2009). Epidemiology of mental disorders in children and adolescents. Dialogues in Clinical Neuroscience, 11(1), 7–20. 19432384 10.31887/DCNS.2009.11.1/krmerikangas PMC2807642

[brb31714-bib-0067] Naaktgeboren, C. A. (2010). Effects of maternal probiotic exposure during pregnancy and lactation on the mother and infant. International Journal of Probiotics & Prebiotics, 5(3), 113–124.

[brb31714-bib-0068] Namakin, K. , Molae, N. , Sharifzadeh, G. , & Javadinia, S. A. (2016). Anxiety and depression in adolescents with abnormal body mass index with normal population. Journal of Fundamentals of Mental Health, 18(6), 357–360.

[brb31714-bib-0069] Pärtty, A. , Kalliomäki, M. , Wacklin, P. , Salminen, S. , & Isolauri, E. (2015). A possible link between early probiotic intervention and the risk of neuropsychiatric disorders later in childhood: A randomized trial. Pediatric Research, 77(6), 823–828. 10.1038/pr.2015.51 25760553

[brb31714-bib-0070] Paul, H. A. , Bomhof, M. R. , Vogel, H. J. , & Reimer, R. A. (2016). Diet‐induced changes in maternal gut microbiota and metabolomic profiles influence programming of offspring obesity risk in rats. Scientific Reports, 6, 20683 10.1038/srep20683 26868870 PMC4751613

[brb31714-bib-0071] Pellow, S. , Chopin, P. , File, S. E. , & Briley, M. (1985). Validation of open: Closed arm entries in an elevated plus‐maze as a measure of anxiety in the rat. Journal of Neuroscience Methods, 14(3), 149–167. 10.1016/0165-0270(85)90031-7 2864480

[brb31714-bib-0072] Perry, W. , Minassian, A. , Lopez, B. , Maron, L. , & Lincoln, A. (2007). Sensorimotor gating deficits in adults with autism. Biological Psychiatry, 61(4), 482–486. 10.1016/j.biopsych.2005.09.025 16460695

[brb31714-bib-0073] Petersen, A. C. (1988). Adolescent development. Annual Review of Psychology, 39, 583–607. 10.1146/annurev.ps.39.020188.003055 3278681

[brb31714-bib-0074] Pirbaglou, M. , Katz, J. , De Souza, R. J. , Stearns, J. C. , Motamed, M. , & Ritvo, P. (2016). Probiotic supplementation can positively affect anxiety and depressive symptoms: A systematic review of randomized controlled trials. Nutrition Research, 36(9), 889–898. 10.1016/j.nutres.2016.06.009 27632908

[brb31714-bib-0075] Principi, N. , & Esposito, S. (2016). Gut microbiota and central nervous system development. Journal of Infection, 73(6), 536–546. 10.1016/j.jinf.2016.09.010 27725185

[brb31714-bib-0076] Prut, L. , & Belzung, C. (2003). The open field as a paradigm to measure the effects of drugs on anxiety‐like behaviors: A review. European Journal of Pharmacology, 463(1–3), 3–33. 10.1016/S0014-2999(03)01272-X 12600700

[brb31714-bib-0077] Ramos, A. , Kangerski, A. L. , Basso, P. F. , Da Silva Santos, J. E. , Assreuy, J. , Vendruscolo, L. F. , & Takahashi, R. N. (2002). Evaluation of lewis and SHR rat strains as a genetic model for the study of anxiety and pain. Behavioural Brain Research, 129(1–2), 113–123. 10.1016/S0166-4328(01)00337-0 11809502

[brb31714-bib-0078] Reis, D. J. , Ilardi, S. S. , Punt, S. E. W. , & Jane, F. (2018). The anxiolytic effect of probiotics: A systematic review and meta‐analysis of the clinical and preclinical literature. PLoS One, 13(6), e0199041 10.1371/journal.pone.0199041 29924822 PMC6010276

[brb31714-bib-0079] Sampson, T. , Debelius, J. , Thron, T. , Janssen, S. , Shastri, G. , Ilhan, Z. … Chesselet, M. F. (2016). Gut microbiota regulate motor deficits and neuroinflammation in a model of parkinson’s disease. Cell, 167(6), 1469–1480. e12 10.1038/s41390-018-0191-9 27912057 PMC5718049

[brb31714-bib-0080] Sarkisova, K. Y. , & Kulikov, M. A. (2006). Behavioral characteristics of wag/rij rats susceptible and non‐susceptible to audiogenic seizures. Behavioural Brain Research, 166(1), 9–18. 10.1016/j.bbr.2005.07.024 16183145

[brb31714-bib-0081] Satokari, R. , Grönroos, T. , Laitinen, K. , Salminen, S. , & Isolauri, E. (2008). Bifidobacterium and lactobacillus dna in the human placenta. Letters in Applied Microbiology, 48(1), 8–12. 10.1111/j.1472-765X.2008.02475.x 19018955

[brb31714-bib-0082] Schneider, M. (2013). Adolescence as a vulnerable period to alter rodent behavior. Cell and Tissue Research, 354(1), 99–106. 10.1007/s00441-013-1581-2 23430475

[brb31714-bib-0083] Schneier, F. R. , Johnson, J. , Hornig, C. D. , Liebowitz, M. R. , & Weissman, M. M. (1992). Social phobia. comorbidity and morbidity in an epidemiologic sample. Archives of General Psychiatry, 49(4), 282–288. 10.1001/archpsyc.1992.01820040034004 1558462

[brb31714-bib-0084] Severance, E. G. , Yolken, R. H. , & Eaton, W. W. (2014). Autoimmune diseases, gastrointestinal disorders and the microbiome in schizophrenia: more than a gut feeling. Schizophrenia Research, 176(1), 23–35. 10.1016/j.schres.2014.06.027 25034760 PMC4294997

[brb31714-bib-0085] Sharafi, S. E. , Garmaroudi, G. , Ghafouri, M. , Bafghi, S. A. , Ghafouri, M. , Tabesh, M. R. , & Alizadeh, Z. (2019). Prevalence of anxiety and depression in patients with overweight and obesity. Obesity Medicine, 17, 100169 10.1016/j.obmed.2019.100169

[brb31714-bib-0086] Sivanathan, S. , Thavartnam, K. , Arif, S. , Elegino, T. , & Mcgowan, P. O. (2015). Chronic high fat feeding increases anxiety‐like behaviour and reduces transcript abundance of glucocorticoid signalling genes in the hippocampus of female rats. Behavioural Brain Research, 286, 265–270. 10.1016/j.bbr.2015.02.036 25721737

[brb31714-bib-0087] Slykerman, R. F. , Hood, F. , Wickens, K. , Thompson, J. , Barthow, C. , Murphy, R. , … Mitchell, E. A. (2017). Effect of, lactobacillus rhamnosus, hn001 in pregnancy on postpartum symptoms of depression and anxiety: A randomised double‐blind placebo‐controlled trial. EBioMedicine, 24, 159–165. 10.1016/j.ebiom.2017.09.013 28943228 PMC5652021

[brb31714-bib-0088] Spear, L. P. (2000). The adolescent brain and age‐related behavioral manifestations. Neuroscience & Biobehavioral Reviews, 24(4), 417–463. 10.1016/S0149-7634(00)00014-2 10817843

[brb31714-bib-0089] Swerdlow, N. R. , Auerbach, P. , Monroe, S. M. , Hartston, H. , Geyer, M. A. , & Braff, D. L. (1993). Men are more inhibited than women by weak prepulses. Biological Psychiatry, 34(4), 253–260. 10.1016/0006-3223(93)90079-S 8399822

[brb31714-bib-0090] Swerdlow, N. R. , & Geyer, M. A. (1998). Using an animal model of deficient sensorimotor gating to study the pathophysiology and new treatments of schizophrenia. Schizophrenia Bulletin, 24(2), 285–301. 10.1093/oxfordjournals.schbul.a033326 9613626

[brb31714-bib-0091] Swerdlow, N. R. , Paulsen, J. , Braff, D. L. , Butters, N. , Geyer, M. A. , & Swenson, M. R. (1995). Impaired prepulse inhibition of acoustic and tactile startle response in patients with huntington's disease. Journal of Neurology, Neurosurgery and Psychiatry, 58(2), 192–200. 10.1136/jnnp.58.2.192 PMC1073317 7876851

[brb31714-bib-0092] Tremlett, H. , Bauer, K. C. , Appel‐Cresswell, S. , Finlay, B. B. , & Waubant, E. (2017). The gut microbiome in human neurological disease: A review. Annals of Neurology, 81(3), 369–382. 10.1002/ana.24901 28220542

[brb31714-bib-0093] Vyas, U. , & Ranganathan, N. (2012). Probiotics, prebiotics, and synbiotics: Gut and beyond. Gastroenterology Research & Practice, 2012(11), 872716 10.1155/2012/872716 23049548 PMC3459241

[brb31714-bib-0094] Walker, R. W. , Clemente, J. C. , Peter, I. , & Loos, R. J. F. (2017). The prenatal gut microbiome: Are we colonized with bacteria *in utero*? Pediatric Obesity, 12, 3–17. 10.1111/ijpo.12217 28447406 PMC5583026

[brb31714-bib-0095] Wall, R. , Marques, T. M. , O’Sullivan, O. , Ross, R. P. , Shanahan, F. , Quigley, E. M. , … Stanton, C. (2012). Contrasting effects of bifidobacterium breve ncimb 702258 and bifidobacterium breve dpc 6330 on the composition of murine brain fatty acids and gut microbiota. American Journal of Clinical Nutrition, 95(5), 1278–1287. 10.3945/ajcn.111.026435 22492373

[brb31714-bib-0096] Walsh, R. N. , & Cummins, R. A. (1976). The open‐field test: A critical review. Psychological Bulletin, 83(3), 482–504. 10.1037//0033-2909.83.3.482 17582919

[brb31714-bib-0097] Whitakerazmitia, P. M. (2001). Serotonin and brain development: Role in human developmental diseases. Brain Research Bulletin, 56(5), 479–485. 10.1016/S0361-9230(01)00615-3 11750793

[brb31714-bib-0098] Yano, J. M. , Yu, K. , Donaldson, G. P. , Shastri, G. G. , Ann, P. , Ma, L. , … Hsiao, E. Y. (2015). Indigenous bacteria from the gut microbiota regulate host serotonin biosynthesis. Cell, 161(2), 264–276. 10.1016/j.cell.2015.02.047 25860609 PMC4393509

